# The ethyl acetate fraction of corn silk exhibits dual antioxidant and anti-glycation activities and protects insulin-secreting cells from glucotoxicity

**DOI:** 10.1186/s12906-016-1382-8

**Published:** 2016-11-03

**Authors:** Chia-Chuan Chang, Wei Yuan, Hsiao-Yuh Roan, Jia-Ling Chang, Hsiu-Chen Huang, Yu-Ching Lee, Huey Jen Tsay, Hui-Kang Liu

**Affiliations:** 1 School of Pharmacy, College of Medicine, National Taiwan University, Taipei, Taiwan, ROC; 2 Department of Psychiatry, Cheng Hsin General Hospital, Taipei, Taiwan, ROC; 3 Department of Life Sciences and Institute of Genome Sciences, National Yang-Ming University, Taipei, Taiwan, ROC; 4 Division of Basic Chinese Medicine, National Research Institute of Chinese Medicine, Ministry of Health and Welfare, Taipei, Taiwan, ROC; 5 Department of Applied Science, National Hsinchu University of Education, Hsinchu, Taiwan, ROC; 6 The Center of Translational Medicine, Taipei Medical University, Taipei, Taiwan, ROC; 7 The Ph.D. Program for Medical Biotechnology, College of Medical Science and Technology, Taipei Medical University, Taipei, Taiwan, ROC; 8 Institute of Neuroscience, Brain Research Centre, National Yang-Ming University, Taipei, Taiwan, ROC; 9 Ph.D Program for the Clinical Drug Discovery from Herbal Medicine, College of Pharmacy, Taipei Medical University, Taipei, Taiwan, ROC

**Keywords:** Stigmata Maydis (corn silk), Glucotoxicity, Methylglyoxal, Advanced glycation end products, Reactive oxygen species, β-cell failure

## Abstract

**Background:**

In this study, we aimed to develop a Stigmata Maydis (corn silk) fraction with dual bio-activities against oxidative stress and protein glycation to protect β-cells from diabetes-induced failure.

**Methods:**

Corn silk fractions were prepared by partition and chemically characterised by thin-layer chromatography. Free radical scavenging assay, glycation assay, and cell-based viability test (neutral red) were employed to decide the best fraction. Cell death analysis was executed by annexin V/ Propidium iodide staining. Cell proliferation was measured by WST-1. Finally, β-cell function was evaluated by β-cell marker gene expression (RT-PCR) and acute insulin secretion test.

**Results:**

Four corn silk fractions were prepared from an ethanolic crude extract of corn silk. In vitro assays indicate ethyl acetate fraction (YMS-EA) was the most potent fraction. YMS-EA also attenuated the hydrogen peroxide- or methylglyoxal-induced induction of reactive oxygen species, reduction of cell viability, and inhibition of cell proliferation. However, YMS-EA was unable to prevent hydrogen peroxide-induced apoptosis or advanced glycation end-products-induced toxicity. Under hyperglycemic conditions, YMS-EA effectively reduced ROS levels, improved mRNA expression of insulin, glucokinase, and PDX-1, and enhanced glucose-stimulated insulin secretion. The similarity of bioactivities among apigenin, luteolin, and YMS-EA indicated that dual activities of YMS-EA might be derived from those compounds.

**Conclusions:**

We concluded that YMS-EA fraction could be developed as a preventive food agent against the glucotoxicity to β-cells in Type 2 diabetes.

## Background

Maize is known as corn which is widely grown in the Americas. In the United States alone, approximately 332 million metric tons of corn are grown annually [1]. Corn silk (Stigmata Maydis) is the female part of the corn. Although corn silk is often processed as agriculture waste, it is actually consumed as tea or regarded as an herb in traditional medicine. The main medicinal property of corn silk is to promote fluid excretion and reduce swelling [2]. In addition to its use as a natural diuretic, pharmacological studies of corn silk revealed antioxidant and anti-glycation activities that are used in diabetes, nephritis, or hypertension therapy [3]. In terms of chemical constituents related to these bio-activities, flavonoids such as luteolin, formononetin and apigenin are identified from Stigmata Maydis and their antioxidant properties have been illustrated [4–7]. Additionally, polysaccharides of Stigmata Maydis have been shown to reduce blood glucose and protein glycation in diabetic mice [8, 9].

Under physiological conditions, reactive oxygen species (ROS) and protein glycation are important molecules and essential biochemical events in the human body. However, excessive ROS or accumulated advanced glycation end-products (AGEs) may lead to tissue damage and aging [10]. For instance, diabetes is a complex metabolic disease with hyperglycaemia resulting from either insulin deficiency (Type 1 diabetes) or impaired insulin action and insulin secretory function (Type 2 diabetes) [11]. When diabetes is not well controlled, chronic hyperglycaemia leads to the progression of various diabetic complications via oxidative stress and AGE formation [12].

The loss of β-cell function and mass observed in uncontrolled diabetes may also be owing to glucotoxicity. Due to low levels of antioxidant enzymes, pancreatic β-cells are susceptible to oxidative stress via the production of excessive ROS under hyperglycaemic conditions [13]. As a result, glucotoxicity causes insulin secretory dysfunction and increased β-cell apoptosis, thus initiating a vicious cycle for glycaemic control [14, 15]. Moreover, low-level inflammation and additional oxidative stress from AGEs may affect β-cell proliferation and survival, thereby promoting β-cell failure [16].

Using corn silk constituents that possess both antioxidant and anti-glycation bio-activities, we aimed to generate a corn silk extract fraction that combines both activities to provide protective effects against glucotoxicity in insulin-secreting cells.

## Methods

### Extraction and partition of Stigmata Maydis

**Fig. 1:**
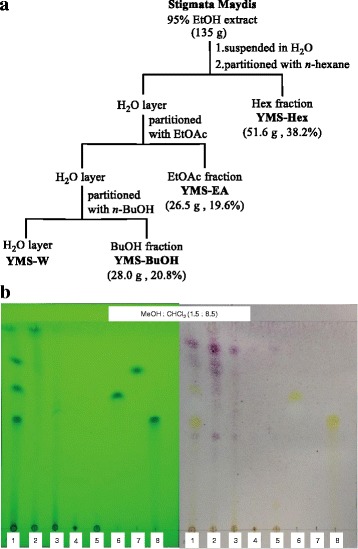
Four YMS Fractions (YMS-Hex, −BuOH, −EA, −W) partitioned from the ethanol extract of Stigmata Maydis (YMS) with chemical characterisation by thin-layer chromatography (TLC). **a** The scheme of the preparation process for four YMS fractions from corn silk (Stigmata Maydis) crude extract. The estimated dry weight and yield of each fraction (except for YMS-W) are listed in parentheses. **b** A representative TLC chromatography of mixed sample (Mix; lane 1), four YMS fractions (−Hex, −EA, −BuOH, −W; lane 2–5), and three flavonoid compounds (apigenin, formononetin, luteolin; lane 6–8)

Stigmata Maydis was purchased from a Fu-Ji Chinese Traditional Medicine Store in Taipei on November 2008. Voucher specimens were deposited with the Herbarium of the National Research Institute of Chinese Medicine (NHP-00351). The ethanol extract (YMS) was made by extracting the dried material of Stigmata Maydis (6 kg) with 100 L of 95 % ethanol at 55 °C for 7 h. Each fraction was made by partitioning the water suspended YMS (135 g) with corresponding solvents, such as *n*-hexane (Hex), ethyl acetate (EA), *n*-butanol (BuOH), to yield YMS-Hex, −BuOH, −EA, and -W fractions, respectively (Fig. 1a). Each YMS fraction and three flavonoid compounds, including apigenin (A), formononetin (F), and luteolin (L), were applied on a Merck thin-layer chromatography plate (Silica gel 60 F_254_, 0.25 mm) (Darmstadt, Germany) under the development in a mobile phase of CHCl_3_ and MeOH (8.5:1.5). Afterwards, separated spots were sprayed with anisaldehyde spray reagent and detected after ultraviolet absorption at 254 nm and 365 nm.

### ABTS free radical scavenging assay

2,2′-azino-*bis* (3-ethylbenzthiazoline-6-sulfonic acid) (ABTS) was used for the measurement of antioxidant activity. Briefly, a reaction mix consisting of potassium persulfate (2.45 mM) in ABTS solution (7 mM) was prepared and kept in the dark at room temperature for at least 16 h before use. The intensively-coloured ABTS^˙+^ solution was then diluted with 0.01 M phosphate buffered saline (PBS) to give a pH of 7.4 with an absorbance of 0.70 at 734 nm. The Stigmata Maydis fractions were diluted 100× with the ABTS^˙+^ solution to a total volume of 1 ml. Absorbance was measured at 6 min after the addition of test reagents. A negative control was made with PBS instead of ABTS^˙+^ solution. The % inhibitions by different concentrations of samples were calculated according to the following equation: $\left[1-\right({\mathrm{Abs}}_{\mathrm{sample}+{\mathrm{ABTS}}^{\dot{\mkern6mu}+}\mathrm{solution}}/\ {\mathrm{Abs}}_{{\mathrm{ABTS}}^{\dot{\mkern6mu}+}\mathrm{solution}}\left)\times 100\right]$ [17].

### Bovine serum albumin (BSA)-methylglyoxal (MG) assay and AGE preparation

This assay was used to evaluate protein glycation, and BSA fluorescence levels were measured. Briefly, BSA (10 mg/ml) was non-enzymatically glycated via incubation in 1 M PBS, pH 7.4, at 37 °C for 7 days in the presence of 1 mM MG and 3 mM sodium azide. The Stigmata Maydis fractions were tested at concentrations of 0.01, 0.02, 0.05, 0.1, and 1.0 mg/ml. Fluorescence of the samples was measured at the excitation and emission wavelengths of 335 and 385 nm, respectively, versus a blank containing the protein and MG. The % inhibition by different concentrations of samples was calculated according to the following equation: [1 − (F_sample + BSA + glucose_ ‐ F_sample + BSA_/ F_BSA + glucose_ ‐ F_BSA_)] × 100. Aminoguanidine (AG) was used as a positive control.

The reactant under control condition was collected to generate AGEs through the dialysis and lyophilisation process. Products were kept at −80 °C for cell-based studies.

### Cell culture

The clonal rat pancreatic β-cell line (BRIN-BD11) was kindly provided by prof. PR Flatt at Univiersity of Ulster, Coleraine, UK and routinely grown as a monolayer in culture dishes at 37 °C under 5 % CO_2_/air with 90 % humidity. Cells were maintained in RPMI 1640 medium containing 10 % foetal bovine serum and 5 % penicillin and streptomycin mixture.

### Cell viability assay (neutral red)

The cell viability assay was performed as previously described [18]. Briefly, at the end of cell treatments, the medium was replaced with the neutral red solution and incubated for another 2 h. Quantification of the uptake of the neutral red by functional lysosomes in cells was spectrophotometrically measured at 540 nm.

### Cell proliferation assay (WST-1)

The WST-1 cell proliferation assay was performed according to the manufacture’s protocol (Cayman Chemical). Briefly, cells were seeded on 96-well plates and the culture medium was replaced with various conditioned medium for 48 h. At the end of treatment, the WST-1 reagent was added and incubated for another 2 h. Finally, the plate was directly measured for absorbance at 450 nm.

### Spectrofluorometric measurement of intracellular ROS

Intracellular ROS were measured by the CM-H_2_DCFDA assay. Cells were cultured at 37 °C with various conditions which were described in figure legends. After 24 h, medium was replaced with the peroxide sensitive fluorescent probe, 5,6-dicarboxy-2,7-dichlorodihydro fluorescein diacetate (carboxy-H_2_DCFDA; 20 μM), for an additional 30 min at 37 °C. The cells were then solubilised with 1 % SDS and 5 mM Tris HCl (pH 7.4). The fluorescence intensity of the lysate was determined using a spectrofluorometer with excitation and emission wavelengths of 495 nm and 517 nm, respectively.

### Flow cytometry with annexin V/Propidium iodide (PI) staining

BRIN-BD11 cells were treated as mentioned above. Afterwards, they were trypsinised, pelleted, and resuspended in culture medium at a concentration of 1 × 10^6^ cells/ml. After transferring 0.5 ml of the cell suspension to a new tube, 10 μl media binding reagent and 1.25 μl annexin V-FITC were added. Following gentle vortexing, the mixture was incubated for 15 min at room temperature in the dark. After centrifuging at 1000xg for 5 min at room temperature, media was removed and 0.5 ml of cold 1× binding buffer and 10 μl propidium iodide were added. Following gentle vortexing, the sample was analysed on the flow cytometer within a 1 h period. The percentages of apoptotic and necrotic cells for each sample were estimated [19].

### Gene expression analysis

BRIN-BD11 cells were seeded on 6 cm-dish (5 × 10^5^ cells/dish) and cultured under the condition described in Figure legends. At the end of experiments, total RNA were extracted and reverse transcribed. 50 ng of complementary (c)DNA of each sample was used for later polymerase chain reaction (PCR). Respective primer sequence, annealing temperature, and size of PCR product of each gene was listed below. Beta-actin: For 5′-CGTAAAGACCTCTATGCCAA-3′ and Rev 5′-AGCCATGCCAAATGTGTCAT-3′; 57 °C; 349b.p. Glucokinase: For 5′-AAGGGAACTACATCGTAGGA-3′ and Rev 5′-CATTGGCGGTCTTCATAGTA-3′; 57 °C; 130b.p. pancreatic and duodenal homeobox-1 (PDX-1): For 5′-CTCGCTGGGAACGCTGGAACA-3′ and Rev 5′-GCTTTGGTGGATTTCATCCACGG-3′; 55 °C; 225b.p. Insulin: For 5′-TGCCCAGGCTTTTGTCAAACAGCACCTT-3′ and Rev 5′-CTCCAGTGCCAAGGTCTGAA-3′; 52 °C; 187b.p. PCR products were separated by ethidium bromide stained gel electrophoresis, visualized, photographed with a digital camera, and quantified with Genetools 3.06 (Syngene, Frederick, MD, USA) [20].

### Insulin secretion

BRIN-BD11 cells were plated on 24-well plates (0.5 × 10^5^ cells/well) and incubated for 48 h with media containing 5.6 or 30.0 mM glucose. Then, after 1 h of pre-incubation with 1.1 mM glucose, cells were challenged either with 1.1 mM or with 16.7 mM glucose in Krebs-Ringer Bicarbonate Buffer for 20 min. The media were collected for insulin determination. Insulin concentrations were quantified by the Homogeneous Time-Resolved Fluorescence (HTRF) insulin assay and normalized to a million of total cell numbers [21].

### Statistical analysis

Data were presented as mean ± standard error of the mean. Statistical analyses were performed using GraphPad Prism (GraphPad, CA, USA). Single parameter-based comparisons were obtained from the unpaired student’s *t*-test. *P* values less than 0.05 and 0.01 were considered to be significant. Multiparametric comparisons were performed using one-way ANOVA, followed by post-hoc analyses by Tukey’s HSD protected least significant difference.

## Results

### Chemical characterization of four YMS fractions partitioned from an ethanolic crude extract of corn silk

As shown in Fig. 1b, all fractions and the three flavonoid compounds were individually developed on a single TLC plate. In addition, all samples were pooled and co-spotted on the same position on the 1^st^ lane (Mix). According the polarity of the solvents for extraction, the *R*_f_ values of all the detected compounds were distributed among the ranges of Hex (0.71 to 0.91), EA (0.52–0.71), BuOH (0.01 to 0.52) and H^2^O (0.0 to 0.01) fractions. The *R*_f_ values for two (apigenin and luteolin) of the three flavonoid compounds (apigenin: 0.66; formononetin: 0.74; luteolin: 0.57) were in the range of the YMS-EA fraction.

### The ethyl acetate fraction (YMS-EA) most potently scavenges free radicals in vitro and protects against effects of H_2_O_2_ on β-cells

All fractions exhibited dose-dependent free radical scavenging effects (Fig. 1a). However, the effect of YMS-EA was superior to that of YMS-Hex, −BuOH, and -W (*p* < 0.001), as it provided 75 % inhibition at a concentration of 100 μg/ml. Therefore, we chose YMS-EA as the major fraction and compared its effects with those of other test agents. First, to compare the protective effects of YMS fractions and reference drugs on H_2_O_2_-mediated ROS production and β-cell death, BRIN-BD11 cells were treated with H_2_O_2_ (125 μM) in the presence of YMS fractions (100 μg/ml), AG (2 mM), metformin (Met; 100 μM), or trolox (Trox; 100 μM) for 24 h.

**Fig. 2:**
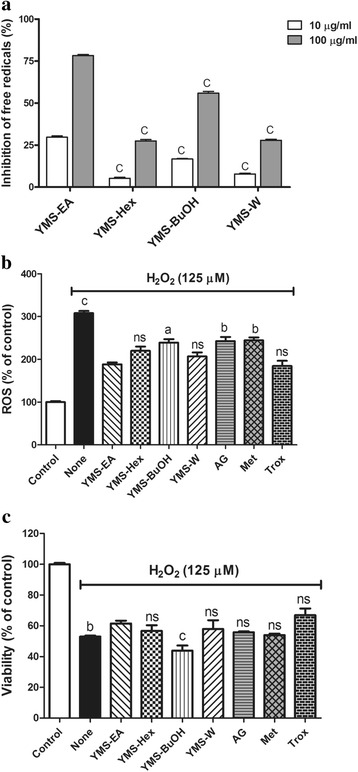
Comparison of YMS fractions and reference drugs for bio-activities against ABTS- and H_2_O_2_-mediated free radical generation and H_2_O_2_- induced cell death. **a** Free radical scavenging activities of YMS fractions. Data are mean ± standard error of the mean (SEM); *n* = 3. ^c^ *p* < 0.001 versus the corresponding YMS-EA group. **b** Inhibitory effects of YMS fractions (100 μg/ml), aminoguanidine (AG; 2 mM), metformin (Met; 100 μM), and trolox (Trox; 100 μM) on H_2_O_2_ (125 μM)-induced ROS levels in BRIN-BD11 cells after 24 h. Data are mean ± SEM (*n* = 5). ^a^ *p* < 0.05, ^b^ *p* < 0.01, ^c^ *p* < 0.001 versus the YMS-EA group. **c** Viability of BRIN-BD11 cells that were treated with H_2_O_2_ (125 μM) for 24 h in the presence of YMS fractions, AG, Met, and Trox. Data are mean ± SEM (*n* = 5). ^b^ *p* < 0.01 and ^c^ *p* < 0.001 versus the YMS-EA group

As shown in Fig. 2b, there is nearly a three-fold increase in ROS levels in H_2_O_2_-treated BRIN-BD11 cells. The presence of YMS-EA significantly decreased ROS levels in H_2_O_2_-treated BRIN-BD11 cells. Comparing YMS-EA with other test agents, only YMS-Hex, YMS-W, and Trox exhibited similar activities. In addition, there was a 50 % reduction in the viability of H_2_O_2_-treated BRIN-BD11 cells after 24 h (Fig. 2c). YMS-EA significantly improved the cell viability of H_2_O_2_-treated BRIN-BD11 cells. YMS-Hex, YMS-W, AG, Met, and Trox provided similar protective effects.

### YMS-EA and reference drugs attenuate the effects of acute H_2_O_2_ treatment on proliferation in BRIN-BD11 cells

**Fig. 3:**
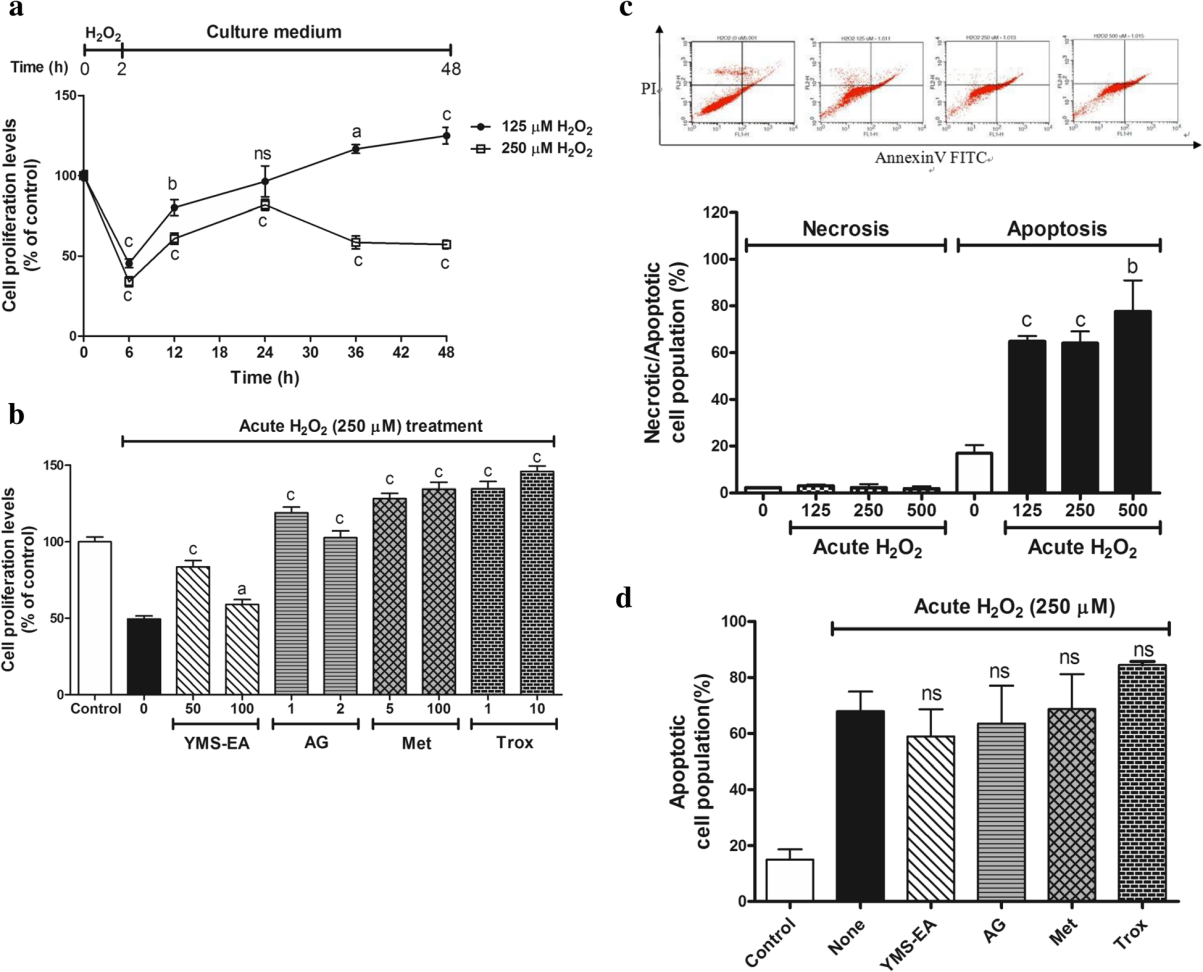
Evaluation of YMS-EA and reference drugs against acute H_2_O_2_ challenge mediated anti-proliferation and apoptosis in BRIN-BD11 cells. **a** BRIN-BD11 cells were treated with H_2_O_2_ for 2 h, and then the medium was replaced with culture medium. The cell proliferation assay (WST-1) was performed at 0, 6, 12, 24, 36, and 48 h. Data are mean ± SEM (*n* = 8). ^a^ *p* < 0.05, ^b^ *p* < 0.01, ^c^ *p* < 0.001 versus time 0. **b** Cell proliferation activity at the end of 48 h in the culture medium with YMS-EA (100 μg/ml), AG (2 mM), Met (100 μM) or Trox (100 μM) after acute H_2_O_2_ (250 μM) challenge. Data are mean ± SEM (*n* = 8). ^a^ *p* < 0.05, ^b^ *p* < 0.01, ^c^ *p* < 0.001 versus no treatment after H_2_O_2_ challenge. **c** The population of necrotic or apoptotic cells was evaluated at 24 h after acute H_2_O_2_ (250 μM) challenge. Data are mean ± SEM (*n* = 4). ^b^ *p* < 0.01, ^c^ *p* < 0.001 versus the corresponding population with no treatment after H_2_O_2_ challenge. **d** Apoptotic cell population in the presence of test agents at 24 h after H_2_O_2_ (250 μM) challenge. Data are mean ± SEM (*n* = 4)

In terms of the impact of acute H_2_O_2_ treatment on cell proliferation, BRIN-BD11 cells were transiently treated with H_2_O_2_ for 2 h, and proliferation was monitored at 24 h post-treatment. The cell proliferation rate of BRIN-BD11 cells treated with H_2_O_2_ (125 or 250 μM) dropped over 50 % at 6 h post-treatment (Fig. 3a). At 24 h post-treatment, the cell proliferation ratio of BRIN-BD11 cells that were treated with the lower concentration of H_2_O_2_ (125 μM) returned to original levels. After 48 h, there was a 50 % reduction in the proliferation of BRIN-BD11 cells that were treated with higher concentration of H_2_O_2_ (250 μM). Therefore, the effects of YMS-EA and reference drugs were tested in the presence of 250 μM H_2_O_2_ .

After acute H_2_O_2_ challenge, the treatment with YMS-EA or reference drugs insufficiently improved cell proliferation (Fig. 3b). YMS-EA had a better effect at 50 μg/ml compare with that at 100 μg/ml. However, all of the reference drugs performed better than 100 μg/ml YMS-EA (*p* < 0.001).

According to the cell death analysis, the transient treatment of BRIN-BD11 cells with H_2_O_2_ for 2 h for BRIN-BD11 cells caused late apoptosis in a dose-dependent manner at 24 h post-treatment. The apoptotic cell population reached 70 % after 24 h when BRIN-BD11 cells were transiently challenged with 250 μM H_2_O_2_ (Fig. 3c). Under this condition, treatment with YMS-EA or reference drugs had no effect (Fig. 3d).

### YMS-EA treatment ameliorates MG-mediated glycation, ROS production, and cell death

**Fig. 4:**
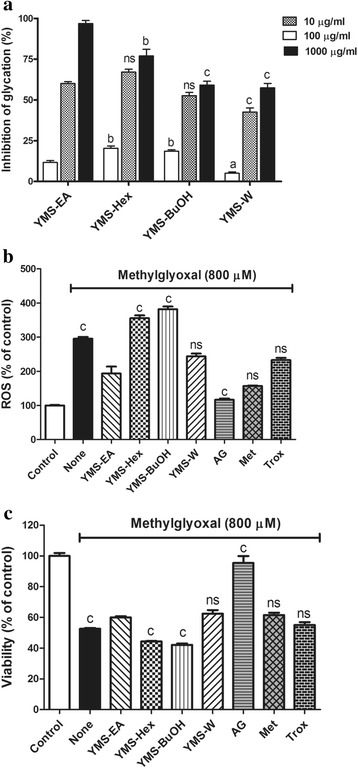
Comparison of YMS fractions and reference drugs for bio-activities against methylglyoxal-mediated in vitro glycation, ROS elevation, and viability reduction. **a** Anti-glycation activities of YMS fractions (100 μg/ml), AG (2 mM), Met (100 μg/ml), and Trox (100 μg/ml). Data are mean ± SEM; *n* = 3. ^a^ *p* < 0.05, ^b^ *p* < 0.01, ^c^ *p* < 0.001 versus the corresponding YMS-EA group. **b** Inhibitory effects of YMS fractions and reference drugs on methylglyoxal (800 μM)-induced reactive oxygen species (ROS) levels after 24 h. Data are mean ± SEM (*n* = 5). ^c^ *p* < 0.001 versus the YMS-EA group. **c** Protective effects of YMS fractions and reference drugs on methylglyoxal (800 μM)-mediated cell death after 24 h. Data are mean ± SEM (*n* = 5). ^c^ *p* < 0.001 versus the YMS-EA group

The anti-glycation activity was determined by the production efficiency of fluorescent AGEs, which were generated by co-incubating BSA and MG (Fig. 4a). All YMS fractions inhibited the formation of AGEs in a dose-dependent manner. At low concentrations (10 μg/ml), the inhibitory effect of YMS-EA was less effective than that of YMS-Hex and -BuOH. However, at high concentrations (1000 μg/ml), YMS-EA demonstrated the most effective anti-glycation activity when compared with YMS-Hex, −BuOH, and -W (*p* < 0.01, *p* < 0.001, and *p* < 0.001, respectively).

As shown in Fig. 4b, MG (800 μM)-treated BRIN-BD11 cells exhibited three-fold increase in ROS levels after 24 h. Treatment with 100 μg/ml YMS-EA significantly reduced ROS production. Similar effects were observed with YMS-W, Met, and Trox. AG appeared to be the most potent anti-glycation agent. Furthermore, after BRIN-BD11 cells were treated with 800 μM MG for 24 h, viability decreased by nearly 50 % (Fig. 4c). In the presence of YMS-EA, there was a significant increase in the viability of BRIN-BD11 cells. Similar effects were observed with YMS-W, Met, and Trox. It is important to note that AG treatment elicited a nearly 100 % protective effect in BRIN-BD11 cells.

### YMS-EA treatment has no protective effects on AGE-mediated cell death and anti-proliferation in BRIN-BD11 cells

**Fig. 5:**
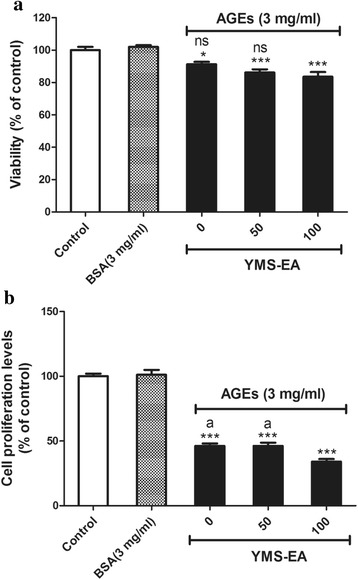
Advanced glycation end product (AGE)-mediated cell death and anti-proliferation effects on BRIN-BD11 cells were unaffected by YMS-EA treatment. Effects of a 48-h cell culture with AGEs on cell viability (**a**) and proliferation (**b**) in the presence or absence of Y2S-EA were measured. Data are mean ± SEM (*n* = 8). ^***^ *p* < 0.001 versus the BSA group

The presence of AGEs (3 mg/ml) in the culture medium significantly reduced viability at 48 h post-treatment (*p* < 0.05) (Fig. 5a). There was no difference in cell viability under this condition in the presence of YMS-EA (50 or 100 μg/ml). Additionally, treatment with 3 mg/ml AGEs had a potent anti-proliferation effect on BRIN-BD11 cells (*p* < 0.001). Surprisingly, instead of providing beneficial effects on cell proliferation, the presence of YMS-EA (100 μg/ml) significantly worsened cell proliferation (*p* < 0.05).

### Addition of YMS-EA attenuates the hyperglycaemia-induced elevation of ROS, reduction of β-cell marker genes, and impairment of glucose responsiveness in BRIN-BD11 cells

**Fig. 6:**
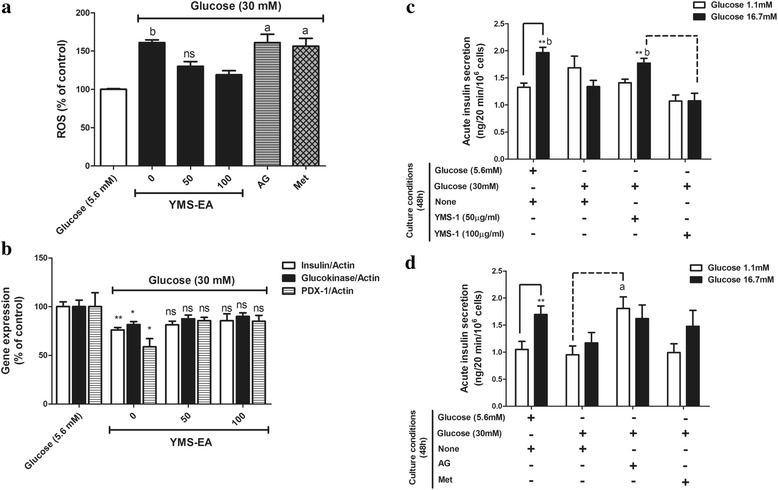
Beneficial effects of YMS-EA on BRIN-BD11 cells in a high-glucose (30 mM) medium induced the generation of free radicals, the reduction of β-cell marker genes, and the impairment of glucose-induced insulin secretion. **a** Effects of YMS-EA, AG (2 mM), and Met (100 μM) on ROS levels in BRIN-BD11 cells cultured in high-glucose medium for 48 h. Data are mean ± SEM (*n* = 6). ^a^ *p* < 0.05 and ^b^ *p* < 0.01 versus the YMS-EA (100 μg/ml) group. **b** Effects of YMS-EA on the gene expression of insulin/glucokinase/ pancreatic and duodenal homeobox-1 (PDX-1) in BRIN-BD11 cells cultured in high-glucose medium for 48 h. Data are mean ± SEM (*n* = 4). ^*^ *p* < 0.05 and ^**^ *p* < 0.01 versus the control culture condition (5.6 mM glucose). **c** Effects of YMS-EA on the glucose-responsiveness of BRIN-BD11 cells cultured in high-glucose medium for 48 h. Data are mean ± SEM (*n* = 8). **p < 0.01 versus the 1.1 mM glucose group-under the same culture condition. ^b^ *p* < 0.01 versus the corresponding YMS-EA (100 μg/ml) group. **d** Effects of AG and Met on the glucose-responsiveness of BRIN-BD11 cells cultured in high-glucose medium for 48 h. Data are mean ± SEM (*n* = 6). **p < 0.01 versus the 1.1 mM glucose group-under the same culture condition. ^a^ *p* < 0.05 versus the corresponding condition of None (30 mM Glucose) group

Compared with cells cultured under 5.6 mM glucose, cells cultured under 30 mM glucose exhibited a 1.5-fold increase in ROS levels (Fig. 6a). The presence of YMS-EA significantly suppressed the induction of ROS by 30 mM glucose (*p* <0.01). Both concentrations of YMS-EA were equally effective, whereas none of the reference drugs had ROS scavenging effects.

We further analysed the gene expression of β-cell markers in the presence of 5.6 mM or 30 mM glucose. There was a significant reduction in the mRNA levels of insulin, glucokinase, and pancreatic and duodenal homeobox-1 (PDX-1) (Fig. 6b). Consistently, the presence of YMS-EA attenuated this reduction in the mRNA levels of β-cell markers.

Finally, when BRIN-BD11 cells were cultured under 30 mM glucose for 48 h, the glucose responsiveness of BRIN-BD11 cells was abolished (Fig. 6c). Treatment of 50 μg/ml YMS-EA restored insulin secretory activity in response to 16 mM glucose (*p* < 0.01). However, this beneficial effect did not appear when 100 μg/ml YMS-EA was used. Under the stimulated condition (16.7 mM glucose), the amount of insulin secreted from YMS-EA (50 μg/ml)-treated BRIN-BD11 cells was significantly more (*p* <0.01) than that from YMS-EA (100 μg/ml)-treated cells. In contrast, in Fig. 6d, treatment of AG could only significantly enhanced insulin secretion under basal condition (*p* <0.05) rather than stimulated condition.

### Individual effects of three flavonoid compounds on dual activities and beta-cell protection

**Fig. 7:**
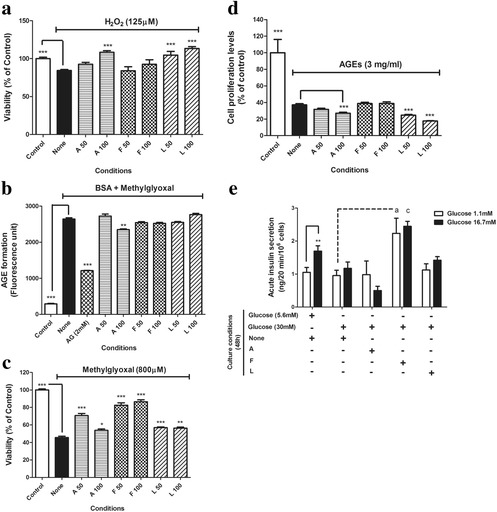
Evaluation of dual activities and beta-cell protective effects of three flavonoids. To explore the activity relationship between YMS-EA and three flavonoids, we repeated following experiments by using three flavonoids (μM). **a** H^2^O^2^- induced cell death. Data are mean ± SEM (*n* = 6). ***p < 0.001 versus the None group. **b** AGE formation assay. Data are mean ± SEM (*n* = 3). ***p < 0.001 versus the None group. **c** Methylglyoxal- induced cell death. Data are mean ± SEM (*n* = 12). *p < 0.05 and ***p < 0.001 versus the None group. **d** AGEs inhibited cell proliferation assay. Data are mean ± SEM (*n* = 8). ***p < 0.001 versus the None group. **e** Glucose stimulated insulin secretion of hyperglycemia damaged cells. Data are mean ± SEM (*n* = 6). **p < 0.01 versus the 1.1 mM glucose group-under the same culture condition. ^a^ *p* < 0.05 and ^c^ *p* < 0.001 versus the corresponding condition of None (30 mM Glucose) group

By employing previous experiments, individual effects of apigenin (A), formononetin (F) and luteolin (L) were examined. In Fig. 7a, only apigenin and luteolin provided a dose-dependent protective effect on H_2_O_2_ –induced bête-cell death. In terms of anti-AGE formation, only apigenin at 100 μM can significantly inhibited AGE formation (*p* <0.01). Furthermore, three flavonoids provided beta-cell protection against methylglyoxal. Formononetin appeared to be superior to other two (Fig. 7c). In terms of AGEs-inhibited cell proliferation, all flavonoids could provide beneficial effects. Instead, addition of apigenin and luteolin worsen the inhibitory effects of AGEs on cell proliferation. Finally, impaired glucose-responsiveness of hyperglycemia-damaged BRIN-BD11 cells was unable to be restored by three flavonoids (Fig. 7d). However, addition of formononetin partially increased basal insulin secretion (Fig. 7e).

## Discussion

Because a large amount of corn silk is treated as agriculture waste after the processing of corn, using a simple preparation process to develop a corn silk fraction with health benefits would promote the utilization of corn silk. In the present study, we aimed to make a fraction with dual bio-activities, including antioxidant and anti-glycation activities. Aminoguanidine, trolox, and metformin are three reference drugs with single or dual activities against oxidative stress and glycation [22–25]. Our in vitro results indicate that the YMS-EA fraction was the most effective fraction. Although the YMS-BuOH fraction was partitioned with *n*-butanol, which has the closest polarity index number compared to ethyl acetate, it exhibited significantly less bio-activity. The *R*_f_ values of apigenin and luteolin were in the range of YMS-EA suggested that both flavonoids may contribute to the dual bioactivity of YMS-EA. Luteolin derivatives were also previously identified from EA fraction [3]. Interestingly, two independent research publications showing that apigenin has activity to scavenge ROS and luteolin intervenes the formation of AGEs support our view [6, 26].

The islets are known to have relatively low levels of antioxidants, and decreases in blood glutathione levels contribute to the accumulation of ROS in the islet during diabetes. Thus, the application of an antioxidation mechanism to protect beta-cell survival and function is a long-accepted concept [27]. The employment of antioxidants is an important strategy for the preservation of β-cell function, as shown in several experimental and clinical studies [28, 29]. Consistent with the above concept, our results demonstrate that YMS-EA possessed free radical scavenging activity and improved BRIN-BD11 cell viability by reducing ROS production in the presence of H_2_O_2_. This activity of YMS-1 was better than that of YMS-BuOH, AG, and Met. In addition, YMS-EA attenuated the anti-proliferative effect of acute H_2_O_2_ treatment on BRIN-BD11 cells. However, the application of reference drugs appeared to be more effective than YMS-EA. In contrast, the presence of YMS-EA and other reference drugs could not effectively prevent BRIN-BD11 cells from previously triggered apoptosis by H_2_O_2_. Therefore, the present study suggests that YMS-EA and reference drugs are more suitable for the prevention or intervention of ROS-induced cell death. Similar to reference drugs, YMS-EA could not rescue H_2_O_2_-induced apoptotic cells. This was possibly because YMS-EA and reference drugs lack of the DNA repair activity against H_2_O_2_-induced DNA damages and subsequent apoptosis [20, 30].

AGEs are generated from the nonenzymatic interaction between protein and carbohydrates and are regarded as important mediators of diabetes-related complications. AGEs also play a role in beta-cell failure in diabetes [16]. MG is a reactive compound that is derived from glucose and fructose metabolism. It is not only is a ROS donor but plays a role in AGE formation [31]. In the current study, a high concentration of YMS-EA was the most effective at inhibiting MG-mediated AGE formation. However, only YMS-EA and YMS-W significantly inhibited MG-induced ROS production and improved cell survival in BRIN-BD11 cells. Among all test agents, AG was the most potent agent for the prevention of MG-mediated cell death. Our results also further indicate that YMS-EA could not provide any protection to enable β-cell survival against AGE-mediated toxicity. Our findings suggest that YMS-EA has a preventive, but not rescue, effect on the loss of β-cell mass and function in diabetes.

Finally, we evaluated whether YMS-EA could prevent glucotoxicity-induced β-cell dysfunction. The excessive entry of glucose has been shown to elicit β-cell injury and increase the nonenzymatic glycation of cellular proteins in animal studies [32, 33]. A β-cell line cultured under high-glucose concentrations exhibits deteriorating outcomes in insulin, glucokinase, and PDX-1 expression [34–37]. Oxidative stress is also known to play an important role in high glucose-mediated β-cell dysfunction [38]. Interestingly, only YMS-EA could effectively reduce the level of ROS under high-glucose conditions. Such effect was associated with the restoration of the expression of important beta-cell marker genes. However, in terms of the insulin secretory function in response to glucose, the improvement in glucose responsiveness by 50 μg/ml YMS-EA treatment disappeared when 100 μg/ml YMS-EA was used. In the future, an optimized dosage for YMS-EA should be carefully examined.

By examining activities of three flavonoids, results actually pointed out similarity between the actions of three flavonoids and YMS-EA. Consistent with the indication that YMS-EA might contain apigenin and luteolin type of flavonoids, both compounds provided strong anti-oxidant effects while apigenin could also prevent AGE formation. Interestingly, apigenin, luteolin, and YMS-EA at high dose worsen AGEs-inhibited cell proliferation. As a result, the dual effects of YMS-EA might be derived from collaboration of those compounds.

## Conclusions

In conclusion, our study provides some basis to support the notion that Stigmata Maydis could be developed as a dietary agent to protect β-cell survival and function against pathological oxidative stress and protein glycation in diabetes.

## Abbreviations

ABTS: 2,2′-azino-bis (3-ethylbenzthiazoline-6-sulfonic acid)
AGEs: Advanced glycation end-products
BSA: Bovine serum albumin
carboxy-H2DCFDA: 5,6-dicarboxy-2,7-dichlorodihydro fluorescein diacetate
HTRF: Homogeneous Time-Resolved Fluorescence
MG: Methylglyoxal
PBS: Phosphate buffered saline
PDX-1: Pancreatic and duodenal homeobox-1
PI: Propidium iodide
ROS: Reactive oxygen species
